# Gene Expression Profiles of Peripheral Blood Monocytes in Osteoarthritis and Analysis of Differentially Expressed Genes

**DOI:** 10.1155/2019/4291689

**Published:** 2019-11-26

**Authors:** Ting Shi, Xiongjie Shen, Ge Gao

**Affiliations:** ^1^Department of Clinical Laboratory, Hunan Provincial People's Hospital, Changsha 410005, Hunan, China; ^2^Department of Spine Surgery, Hunan Provincial People's Hospital, Changsha 410005, Hunan, China; ^3^Department of Clinical Laboratory, Xiangya School of Medicine, Central South University, Changsha 410005, Hunan, China

## Abstract

**Background:**

There is little understanding of the molecular processes involved in the pathogenesis of osteoarthritis, limiting early diagnosis and effective treatment of OA. Use of genechips can provide insights into the molecular pathogenesis of diseases. In this study, determination of gene expression profiles of osteoarthritis peripheral blood mononuclear cells will allow exploration of the molecular pathogenesis of OA and find out more candidate biomarkers and potential drug targets of OA.

**Result:**

A total of 1231 DEGs were screened out including 791 upregulated DEGs and 440 downregulated DEGs. The most significant upregulated DEG was RPL38, which may inhibit chondrocyte differentiation and synthesis of the extracellular matrix. *PIK3CA*, *PIK3CB*, *PIK3CD*, *PIK3R1*, *MAPK14*, *IL1A*, *JUND*, *FOSL2*, and *PPP3CA* were the gene symbols of the osteoclast differentiation pathway which was the most significant pathway enriched by DEGs. However, the MAPK signaling pathway occupied the core position of all the pathways which can regulate apoptosis, cell cycle, wnt signaling pathway, p53 signaling pathway, and phosphatidylinositol signaling system. Furthermore, PI3Ks may regulate IL1A, JUND, FOSL2 and PPP3CA through the MAPK signaling pathway.

**Conclusion:**

These identified DEGs and pathways may be novel biomarkers to monitor the changes of OA and can be a potential drug target for the treatment of OA.

## 1. Background

Osteoarthritis (OA) is a chronic degenerative joint disease characterized by degeneration of articular cartilage, synovium inflammation, imbalance in the synthesis and catabolism of the extracellular matrix of chondrocytes, and the formation of subchondral bone and osteophytes [1]. OA is common in the elderly, especially people older than 65 [2]. It is predominant in heavily loaded joints including the knee, hip, spine, and finger joints and ultimately leads to joint dysfunction [3]. Although there are many various therapies to relieve joint pain and improve joint function, the efficacy of these treatments is limited [4]. Joint replacement surgery can only treat patients at the end stage of OA, and X-ray diagnosis is not informative without visible radiographic changes in joint tissue. There is little understanding of the molecular processes involved in the pathogenesis of OA, limiting early diagnosis and effective treatment of OA. Thus, the identification of sensitive biomarkers and the development of novel drug targets at molecular level are key goals of OA research.

Together with the Human Genome Project and the rapid development of molecular biology technology, high-throughput genechip technology has emerged, allowing the rapid and simultaneous analysis of thousands of gene loci [5]. Use of genechips can provide insights into the molecular pathogenesis of diseases. Currently, gene expression profiles of OA have mainly focused on articular cartilage, subchondral bone, and synovium [6–8], but there has not been comprehensive microarray analysis of blood monocytes. Blood is more accessible than tissue, and blood sample collection is less painful for patients, so the identification of sensitive diagnostic biomarkers of OA in the peripheral blood would be highly valuable for clinical application. Peripheral blood mononuclear cells (PBMCs) participate in the occurrence and development of osteoarthritis by promoting osteoclastogenesis and bone resorption, and inhibiting osteoclast apoptosis and interleukin 1 receptor I (IL-1RI) expression [9]. Monocytes increase the degradation of the extracellular matrix of chondrocytes by promoting the expression of matrix metalloproteinase 13 (MMP13), an enzyme that participates in the degradation of extracellular matrix proteins [10]. Monocytes promote the apoptosis of chondrocytes and ultimately lead to cartilage degeneration [11]. Therefore, determination of gene expression profiles of osteoarthritis PBMCs will allow exploration of the molecular pathogenesis of osteoarthritis and may help identify improved targets for the treatment of osteoarthritis.

In this study, gene expression profiles of osteoarthritis PBMCs were constructed by genechip technology. Differentially expressed genes (DEGs) were screened out by comparing the genechip results of osteoarthritis patients with those of normal controls. To obtain greater insights into the molecular mechanisms of OA, we applied bioinformatics analysis. Gene ontology (GO) analysis and pathway enrichment analysis were performed for DEGs on the Gene-Cloud of Biotechnology Information (GCBI) bioinformatics platform, revealing the core genes and signaling pathways in the pathogenesis of OA. In addition, the network relationships between DEGs and signaling pathways were determined by pathway relation and gene signal network analyses, revealing key players in the molecular pathogenesis of OA.

## 2. Results

### 2.1. Identification of Differentially Expressed Genes

**Figure 1:**
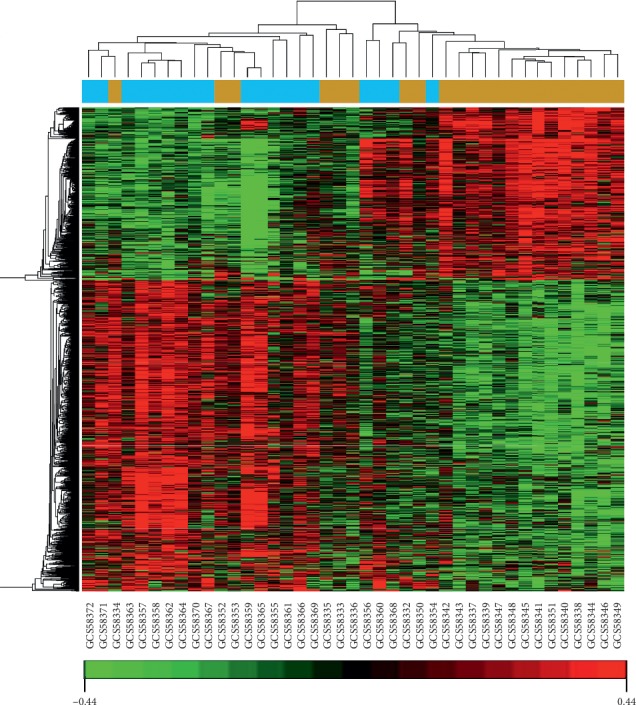
A heatmap. The horizontal axis indicated cluster analysis of groups. Blue represented OA groups, and brown represented control groups. The vertical axis meant cluster analysis of genes. Red represented upregulated DEGs, green represented downregulated DEGs, and black represented the genes expression with no difference. The more the significant difference of gene expression, the brighter the color.

Gene expression profiles of peripheral blood monocytes for OA groups and control groups were compared, revealing 1231 DEGs. Of these genes, 791 were upregulated and 440 were downregulated. We ranked the differentially expressed genes according to the *P* value. The top thirty up- and downregulated DEGs are listed in Table 1. The lowest *P* value of the upregulated DEGs was ribosomal protein L38 (*RPL38*; *P* = 5.30 × 10^−05^), followed by protein phosphatase 3, catalytic subunit, alpha isozyme (*PPP3CA*; *P* = 7.40 × 10^−05^). The most significant downregulated DEG was IKAROS family zinc finger 1 (*IKZF1*; *P* = 1.36*E* − 04), followed by chromosome 7 open reading frame 43 (*C7orf43*; *P* = 1.87*E* − 04). Cluster analysis was performed on the differentially expressed genes, and the result is represented as a heatmap (Figure 1).

### 2.2. GO Analysis and Pathway Enrichment Analysis of DEGs

We next performed GO analysis of the identified DEGs and found 449 biological processes that were significantly enriched. The 30 most enriched GO terms and related DEGs are shown in Table 2. We found that the DEGs were mainly enriched in small molecule metabolic process (GO:0044281, GO:0006468, and GO:0044267), immune process (GO:0045087, GO:0006955, GO:0006954, GO:0019221, and GO:0050900), cell proliferation and apoptosis (GO:0006915, GO:0008283, GO:0043065, and GO:0008219), cell cycle (GO:0000278, GO:0000090, GO:0000087, and GO:0007049), RNA splicing process (GO:0008380 and GO:0000398), and cell adhesion (GO:0030155). DEGs enriched in these biological processes included *interleukin 1*, *alpha* (*IL1A*), *interleukin 6 receptor* (*IL6R*), *interleukin 7 receptor* (*IL7R*), *protein phosphatase 3*, *catalytic subunit*, *alpha isozyme* (*PPP3CA*), *ribosomal protein L38* (*RPL38*), *phosphatidylinositol-4,5-bisphosphate 3-kinase* (*PIK3*), mitogen-activated protein kinase 14 (*MAPK14*), *tumor necrosis factor superfamily*, *member 10* (*TNFSF10*), *tumor necrosis factor superfamily*, *member 13 (TNFSF13)*, *activating transcription factor 2* (*ATF2*), and others. The DEGs mapped to 143 pathways, and the pathway enrichment analysis is partly presented in Table 3. Many of the genes with altered expression participate in osteoclast differentiation, apoptosis, focal adhesion, and cell cycle. In addition, many DEGs are parts of signaling pathways like the MAPK, PI3K-Akt, calcium, T-cell receptor, and wnt and Jak-STAT signaling pathways. *Tyrosine kinase 2* (*TYK2*), *phosphatidylinositol-4*,*5-bisphosphate 3-kinase*, *catalytic subunit alpha* (*PIK3CA*), *phosphatidylinositol-4,5-bisphosphate 3-kinase*, *catalytic subunit beta* (*PIK3CB)*, phosphatidylinositol-4,5-bisphosphate 3-kinase, catalytic subunit delta (*PIK3CD*), *phosphoinositide-3-kinase*, *regulatory subunit 1* (*PIK3R1*), *MAPK14*, *IL1A*, *jun D proto-oncogene* (*JUND*), *FOS-like antigen 2* (*FOSL2*), and *PPP3CA* genes are related to osteoclast differentiation, and *IL-1A*, *JUND*, *PPP3CA*, and *MAPK14* are involved in the MAPK signaling pathway.

### 2.3. Network Analysis

**Figure 2:**
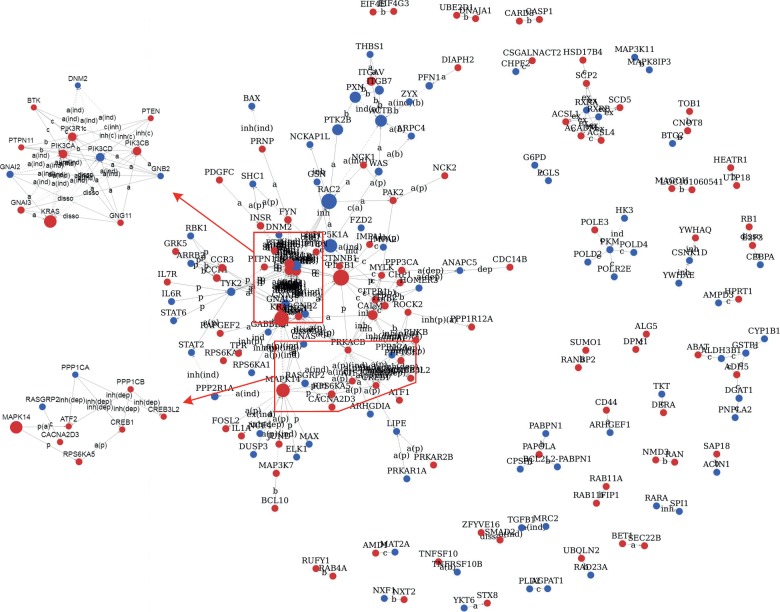
The map of DEG interaction relationship. Red nodes indicate upregulated genes, and blue nodes indicate downregulated genes. The larger the area of the node, the more important the gene in the network. The interaction relationship between genes is expressed by a connection line. The solid line without arrow indicates the interaction between DEGs is not directional, otherwise is directional. The beginning of arrow is the upstream gene, and the end of arrow is the downstream gene. The dashed line with arrow indicates the indirect interaction relationship. The solid line with flat head indicates the inhibitory interaction. The letters on the connection line are abbreviated as the type of interaction. c, compound; a, active; ind, indirect; inh, inhibition.

**Figure 3:**
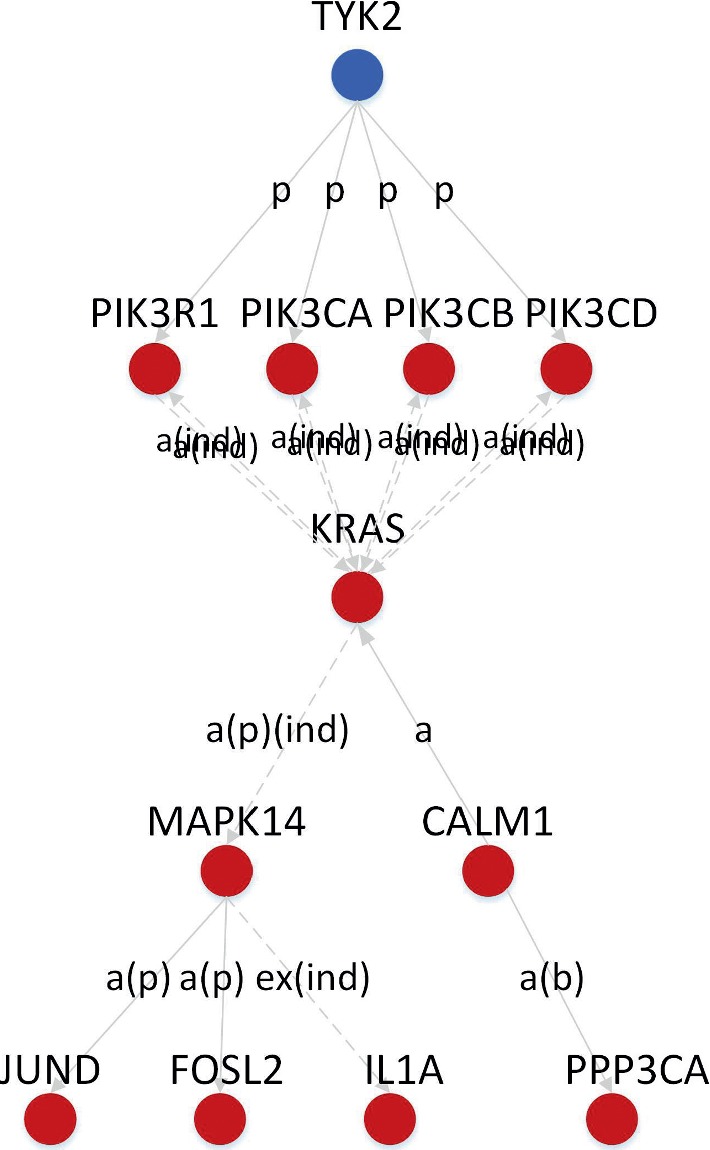
The network relationship of DEGs which enriched in osteoclast differentiation.

The geneSignalNetwork analysis was performed on 1231 DEGs and revealed interactions between 183 DEGs (Figure 2). The results showed that *phospholipase C*, *beta 1 (PLCB1)*, *PIK3CA*, *PIK3CB*, *PIK3CD*, *PIK3R1*, *v-Ki-ras2 Kirsten rat sarcoma viral oncogene homolog* (*KRAS*), *MAPK14*, *protein kinase*, *cAMP-dependent*, *catalytic*, *beta* (*PRKACB*), *phosphatidylinositol-4-phosphate 5-kinase*, *type I*, *alpha* (*PIP5K1A*), *G protein*, *alpha inhibiting activity polypeptide 2* (*GNAI2*), *G protein*, *alpha inhibiting activity polypeptide 3* (*GNAI3*), *calmodulin 1* (*CALM1*), *G protein*, *beta polypeptide 2* (*GNB2*), *G protein*, *gamma 11* (*GNG11*), *beta-actin* (*ACTB*), and *Rac family small GTPase2* (*RAC2*) interact with more than 10 DEGs, making them core genes in the network. The network relationship of DEGs related to osteoclast differentiation, including *PIK3CA*, *PIK3CB*, *PIK3CD*, *PIK3R1*, *MAPK14*, *JUND*, *FOSL2*, *IL1A,* and *PPP3CA*, is shown in Figure 3.

**Figure 4:**
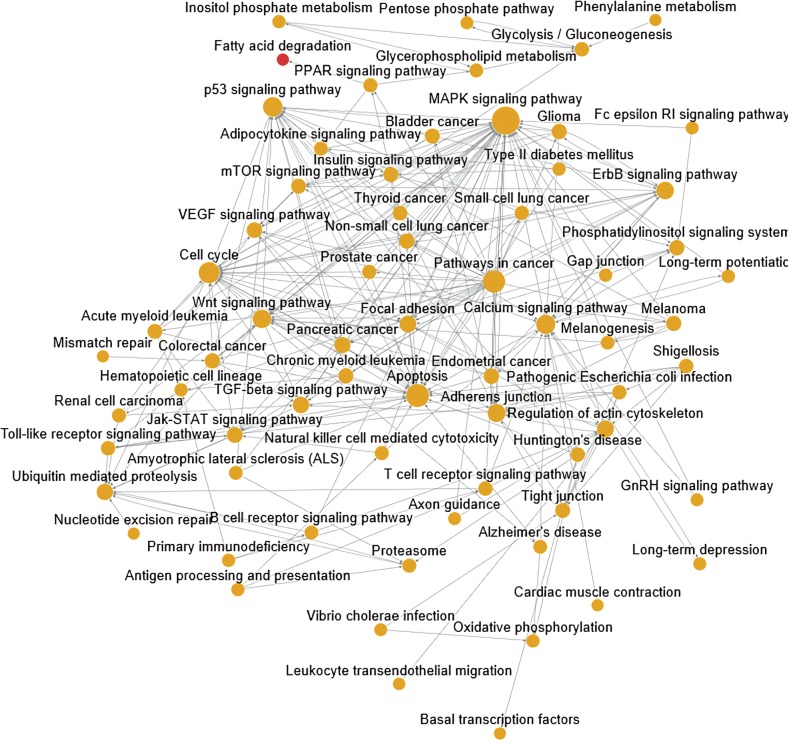
The map of signaling pathway relationship. Yellow nodes indicate the signaling pathways involved by up- and downregulated DEGs. Red nodes indicate the signaling pathways involved by upregulated DEGs. The larger the nodes, the more important they are in the network. The solid line with arrows is used to represent relationship between signaling pathways. The beginning of arrow is the upstream signaling pathway, and the end of arrows is the downstream signaling pathway.

**Figure 5:**
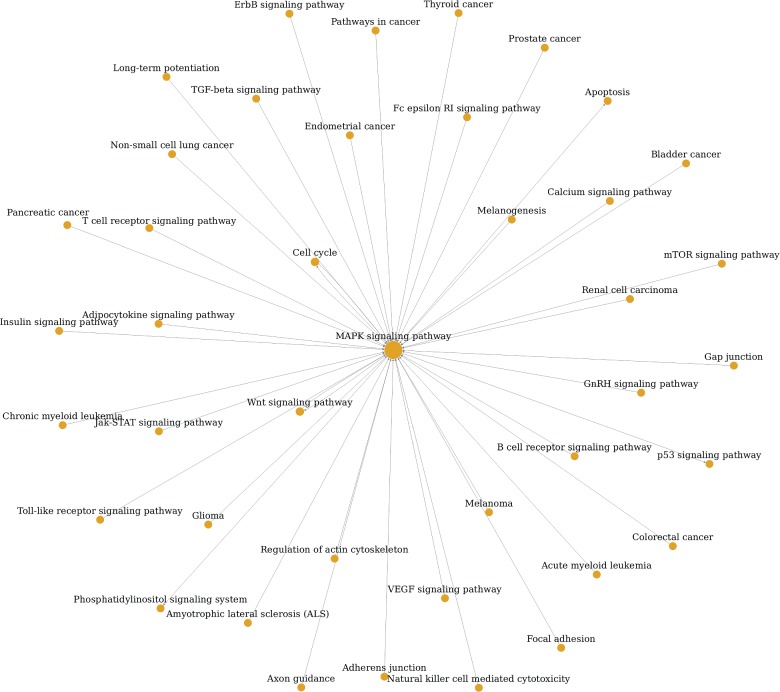
The solar map of all associated signaling pathways of the MAPK signaling pathway.

The pathwayRelationNetwork analysis was also performed and revealed interaction relationships between 68 pathways (Figure 4). The MAPK signaling pathway, apoptosis, pathways in cancer, cell cycle, p53 signaling pathway, calcium signaling pathway, wnt signaling pathway, adherens junction, ErbB signaling pathway, focal adhesion, regulation of actin cytoskeleton, ubiquitin-mediated proteolysis, TGF-beta signaling pathway, and pancreatic cancer interacted with more than 10 pathways, making these core pathways. The most significant pathway in this analysis was the MAPK signaling pathway and its related pathways, as shown in Figure 5. The calcium signaling pathway, cell cycle, wnt signaling pathway, TGF-beta signaling pathway, and VEGF signaling pathway were the source pathways of MAPK signaling pathway. Moreover, MAPK signaling pathway regulated apoptosis, cell cycle, wnt signaling pathway, p53 signaling pathway, and phosphatidylinositol signaling system.

## 3. Discussion

In the present study, the Human Genome U133A genechip from Affymetrix was used to detect the gene expression profiles of PBMCs from 19 patients with osteoarthritis and 22 healthy controls. We identified 1231 DEGs, with 791 upregulated DEGs and 440 downregulated DEGs. The top 10 most significant DEGs according to the *p* value were *RPL38*, *PPP3CA*, *TROVE domain family*, *member 2* (*TROVE2*), *PRP40 pre-mRNA processing factor 40 homolog A* (*PRPF40A*), *centrosomal protein 350 kDa* (*CEP350*), *IKAROS family zinc finger 1* (*IKZF1*), *mitochondrial ribosomal protein S31 (MRPS31)*, *RAB11A*, *member RAS oncogene family* (*RAB11A*), zinc finger CCCH-type containing 14 (*ZC3H14*), and *chromosome 7 open reading frame 43* (*C7orf43*). The most significant upregulated DEG was *RPL38*, which affects processes of gene expression, DNA transcription, gene translation, and cell proliferation. Normal expression of ribosomal protein L29 (RPL29) is essential for chondrocyte proliferation and skeletal growth, and high expression level of RPL29 inhibited chondrocyte terminal differentiation, keeping cells in a state of abnormal proliferation [12]. Altered expression of RPL29 also influenced the rate of extracellular matrix protein synthesis [13]. Green et al. found different expressions of ribosomal protein L10 (RPL10) during endochondral bone development, suggesting effects on cell differentiation before bone mineralization [14]. Given effects of other ribosomal proteins, we hypothesize that ribosomal protein RPL38 may participate in the pathogenesis of OA by inhibiting chondrocyte differentiation and proliferation, decreasing the synthesis of the extracellular matrix.

Osteoclasts are closely related to bone resorption and subchondral bone remodeling processes involved in the pathophysiology of OA [15]. Osteoclasts are large multinucleated cells which originate from hematopoietic precursors of the monocyte-macrophage lineage [16]. Durand et al. found PBMCs from OA patients display increased osteoclastogenesis and bone resorption [9]. Our pathway enrichment analysis of DEGs support previous findings and suggest that the osteoclast differentiation pathway contributes to OA development. *PIK3CA*, *PIK3CB*, *PIK3CD*, *PIK3R1*, *MAPK14*, *IL1A*, *JUND*, *FOSL2*, and *PPP3CA* were the gene symbols of osteoclast differentiation pathway, and these genes showed increased expression in PBMCs of OA patients compared to normal controls. This suggests that mononuclear cells from patients with OA have stronger osteoclast differentiation ability. PIK3CA, PIK3CB, PIK3CD, and PIK3R1 belong to the PI3Ks (phosphoinositide-3-kinases) family [17]. PI3K is not only involved in osteoclast differentiation, activation, and survival but also contributes to osteoclast-mediated bone resorption and in vivo bone homeostasis [18]. PI3K inhibitors such as wortmannin and LY294002 inhibit osteoclast chemotaxis, attachment, and spreading [19]. The PI3K isoform PIK3CB also promotes osteoclast development and bone resorption [20]. JUND and FOSL2 belong to the activator protein-1 (AP-1) transcriptional factor family, which regulates many cellular processes such as differentiation, proliferation, and apoptosis [21]. Fos is a necessary factor for the differentiation of hematopoietic precursor cells into osteoclasts. Mice deficient in Fos (Fos-/-) develop osteopetrosis and show high bone mineral density and defective osteoclast formation. Fos1 transgenic mice showed increased production of osteoclasts and active bone resorption [22]. The JUND/FOSL2 heterodimer upregulates the expression of *Tcirg1* to increase osteoclastogenesis [23]. IL-1A, as a proinflammatory cytokine, can disrupt bone metastasis and stimulate osteoclast resorption activity [24]. PPP3CA, or calcineurin 1, is a serine/threonine-specific phosphatase regulated by Ca^2+^/calmodulin [25]. PPP3C has effects in tumors, cardiovascular system, immune system, and nervous system [26]. The dephosphorylation of PPP3C helps regulate cell proliferation and differentiation in the cytokine-mediated activation of immune cells [27]. Collectively, all these upregulated DEGs associated with osteoclast differentiation may promote the development of OA and have potential to serve as novel biomarkers to monitor changes of OA and are potential drug targets for the treatment of OA.

The most significant pathway revealed by network analysis was the MAPK signaling pathway, with an occupied core position in the network. The MAPK signaling pathway as the source pathway can regulate apoptosis, cell cycle, wnt signaling pathway, p53 signaling pathway, and the phosphatidylinositol signaling system. MAPKs are serine/threonine protein kinases, and activating a protein kinase cascade (MAPKKK-MAPKK-MAPK) allows the transduction of extracellular signals into the nucleus. In this way, the expression of genes related to cell proliferation, differentiation, apoptosis, and stress response can be regulated [28]. The MAPK signaling pathway plays a variety of roles in OA. First, p38-MAPK promotes chondrocyte apoptosis through Fas-mediated apoptotic pathway and activation of ATF-2, caspase-3, and p53 [29]. Second, p38-MAPK induces the expression of MMP13, promotes the degradation of collagen II in the extracellular matrix of chondrocytes, and inhibits the synthesis and secretion of collagen and glycoprotein [30]. Third, p38-MAPK upregulates expression of cyclooxygenase-2 (COX2), prostaglandin E2 (PGE2), and inducible nitric oxide synthase (iNOS), and increases the synthesis of inflammatory mediators, leading to joint swelling, pain, and cartilage destruction [31]. Fourth, p38-MAPK is essential for the differentiation of osteoclast precursor cells into osteoclasts as well as osteoclast maturation and survival [32]. In our analysis of DEGs enriched in the osteoclast differentiation pathway, we found that PIK3CA, PIK3CB, PIK3CD, and PIK3R1 indirectly activate MAPK14, the prototypic member of the p38-MAPK family, through phosphorylation. MAPK14 is a key upstream gene that activates the expression of downstream genes such as IL1A, JUND, FOSL2, and PPP3CA. The network relationships between these genes revealed that PI3Ks may regulate IL1A, JUND, FOSL2, and PPP3CA through the MAPK signaling pathway. However, these regulatory relationships require further experiment to verify.

The results of this study have similarities and differences with published microarray analysis on synovial fluid, synovium membranes, subchondral bone, and cartilage. Firstly, the biological processes enriched by some DEGs are the same, such as immune response and inflammatory response (IL, TNF, TLR, and TGF-*β*), cell cycle (TGF-*β* and SMAD2), cell apoptosis (CASP and BAX). Secondly, high expression of proteinase (ADAMTS10) is same. Thirdly, some signal pathways enriched by DEGs are the same (MAPK signaling pathway, TOLL-like signaling pathway, and TGF-*β* signaling pathway) [6–8]. However, this study found that the main signal pathway enriched by DEGs was osteoclast differentiation, which may be related to the fact that peripheral blood mononuclear cells are precursors of osteoclasts. Only a small portion of the DEGs have the same results as previous tissue microarray because gene expression of PBMCs is affected by several processes. Attur et al. [33] showed that PBMCs can be activated by the inflammatory process of osteoarthritis when they pass through different tissues of the joint. DEGs in PBMCs may reflect the susceptibility of OA from a genetic perspective.

## 4. Conclusions

Gene expression profiles were determined for PBMCs in OA patients and healthy controls. Analysis revealed 1231 DEGs, with 791 upregulated DEGs and 440 downregulated DEGs. The most significant upregulated DEG was RPL38, which may inhibit chondrocyte differentiation and synthesis of the extracellular matrix. *PIK3CA*, *PIK3CB*, *PIK3CD*, *PIK3R1*, *MAPK14*, *IL1A*, *JUND*, *FOSL2*, and *PPP3CA* were the gene symbols of the osteoclast differentiation pathway. These genes promote the development of OA through increasing osteoclastogenesis. PI3Ks may also regulate IL1A, JUND, FOSL2, and PPP3CA through the MAPK signaling pathway. The discovery of these molecular mechanisms associated with the progression of osteoarthritis may contribute to the early diagnosis and treatment of osteoarthritis. Further work should evaluate the utility of these DEGs as biomarkers with diagnostic efficiency, as well as their potential as novel drug targets for the treatment of OA.

## 5. Methods

### 5.1. Research Subjects

The research subjects consisted of 19 OA patients and 22 healthy controls. All the research subjects were collected from Department of Rheumatology and Immunology, the second Xiangya Hospital of Central South University. The symptomatic primary OA patients who met the inclusion criteria were diagnosed by American College of Rheumatology(ACR) standard diagnostic criteria [34], with the Kellgren and Lawrence scoring system rating over grade 1 [35]. The Kellgren and Lawrence scoring system is divided into five grades: grade 0 means normal radiograph; grade 1 means suspicious pathology; grade 2 means small osteophytes and possible joint space narrowing; grade 3 means moderate osteophytes, definite joint space narrowing, and some of subchondral bone sclerosis; and grade 4 means large osteophytes, severe joint space narrowing, and obvious subchondral bone sclerosis. Within the 19 OA patients analyzed, there were 3 OA patients of grade 1, 4 OA patients of grade 2, 6 OA patients of grade 3, and 6 OA patients of grade 4. Exclusion criteria included the diagnosis of RA, osteoporosis, ankylosing spondylitis, neoplastic disease, or any other inflammatory autoimmune diseases. The healthy controls were collected from the same period of physical examination without clinical and radiographic osteoarthritis according to ACR criteria. The age, height, weight, and sex of OA patients and healthy controls were comparable. The research process followed the ethical standards of human experiment in Xiangya Medical College of Central South University and obtained the consent of the research subjects.

### 5.2. Monocyte Extraction and RNA Isolation

We collected fresh peripheral blood from each subject and separated circulating mononuclear cells (MNCs) with UNI-SEP separation tube (Novamed INC, Illinois, USA) according to manufacturer's recommendations. UNI-SEP products are sterile plastic centrifuge tubes containing a solution of 5.6% polysucrose and 9.6% sodium metrizoate. Then, we used monocyte negative isolation kit (Dynal Biotech ASA, Oslo, Norway) to isolate monocytes from MNCs. The antibody mix contains mouse IgG antibodies for CD2, CD7, CD16, CD19, CD56 and CD235a bound the surface of depletion dynabeads, and we discarded beads with unwanted cells and collected untouched monocytes. Then, the monocytes were lysed in TRIzol Reagent (Life Technologies, Bleiswijk, The Netherlands) for 5 min, chloroform was added, and then centrifuged (15 min at 12 000*g*). Transferring the upper aqueous phase to another RNase-free EP tube and adding isopropanol, the tube was placed stably for 10 min and then centrifuged (15 min at 12 000*g*). The sediments (RNA) were washed with 75% ethanol and dissolved in DEPC-treated water. Quality and quantity of the isolated RNA were measured by analyzing samples using the 2100 Bioanalyzer (Agilent Technologies, Amstelveen, The Netherlands) and Nanodrop spectrophotometer.

### 5.3. Microarray Analysis

Total RNA was reverse transcribed to synthesis first-strand cDNA by using a T7-Oligao (dT) Promoter Primer, and then second-strand cDNA was synthesized and purified. Next, in *in vitro* transcription reaction, second-strand cDNA serves as a template performed for complementary RNA (cRNA) amplification and biotin labeling and was cleaned up and fragmented. All the operations were following the instructions of MessageAmp™ Premier RNA Amplification kit (Invitrogen, USA). Subsequently, the biotinylated cRNAs were hybridized to Affymetrix Human Genome U133A genechip overnight at 45°C with rocking. Genechip was then washed and stained according to the protocol of GeneChip® Hybridization, Wash, and Stain kit (Thermo Fisher Scientific, USA). Finally, Affymetrix Gene Array Scanner G2500A scanned the chip, and the scanned images were analyzed by MAS5.0 software.

### 5.4. Screening of DEGs

The image signals of the chip were transformed into the original data of gene expression level by MAS5.0 software. OA group and control group were set up on the GCBI working platform (https://www.gcbi.com.cn/, GMINIX Informatics Ltd. Co, Shanghai, P.R. China). The original data CEL files were imported into the two groups. Using SAM (significance analysis of microarray) R program [36], DEGs between OA group and control group were screened according to the screening criteria of *Q* < 0.05, *P* < 0.05, and fold change > 1.2(*Q* value is the false discovery rate, which represents the expected proportion of false-positive genes in differentially expressed genes. *P* value is adjusted by the *Q* value. When *Q* < 0.05, it can assure lower false discovery rate and higher reliability of results). Hierarchical clustering (a heatmap) was performed with these screened DEGs to identify whether they can make an obvious distinction between OA samples and control samples.

### 5.5. GO Analysis and Pathway Enrichment Analysis of DEGs

We also performed GO (gene ontology) analysis (*P* < 0.05, FDR, false discovery rate < 0.05) and pathway enrichment analysis (*P* < 0.05) for DEGs on the GCBI working platform. In GO analysis, biological processes, cell components, and molecular processes are mainly involved. In the screening of DEGs, more information is provided by biological processes. Therefore, in GO analysis on the GCBI platform, biological processes should be selected as the “analysis type” in parameter setting.

### 5.6. Network Analysis

For DEGs and its associated signaling pathways, we did geneSignalNetwork and pathwayRelationNetwork analysis on the GCBI platform, so the interaction relationship of DEGs and signaling pathways was obtained.

## Figures and Tables

**Table 1:** The top 30 up- and downregulated DEGs in peripheral blood mononuclear cells of patients with OA.

Gene symbol upregulated expression	Accession number	*P* value	Fold change
RPL38	NM_000999	5.30*E* − 05	1.70
PPP3CA	NM_000944	7.40*E* − 05	1.42
TROVE2	NM_001042369	8.10*E* − 05	1.28
PRPF40A	NM_017892	9.50*E* − 05	1.41
CEP350	NM_014810	1.18*E* − 04	1.52
TROVE2	NM_001042369	1.47*E* − 04	1.35
CEP350	NM_014810	1.59*E* − 04	1.41
MRPS31	NM_005830	1.65*E* − 04	1.33
RAB11A	NM_001206836	1.84*E* − 04	1.44
ZC3H14	NM_001160103	1.85*E* − 04	1.35
USP12	NM_182488	1.96*E* − 04	1.38
GTF2H5	NM_207118	2.32*E* − 04	1.37
ITPR1	NM_001099952	2.45*E* − 04	1.35
MTF2	NM_001164391	2.54*E* − 04	1.44
CLCN3	NM_001243372	2.59*E* − 04	1.31
C6orf62	NM_030939	2.94*E* − 04	1.56
MOSPD1	NM_019556	3.25*E* − 04	1.25
PIGF	NM_002643	3.54*E* − 04	1.43
PREPL	NM_001042385	3.89*E* − 04	1.39
THUMPD1	NM_017736	4.08*E* − 04	1.37
CEP57	NM_001243776	4.13*E* − 04	1.41
PHACTR2	NM_001100164	4.41*E* − 04	1.43
ADAMTS10	NM_001110	4.52*E* − 04	1.28
E2F3	NM_001243076	4.53*E* − 04	1.41
ING3	NM_019071	4.71*E* − 04	1.49
MYCBP2	NM_015057	4.75*E* − 04	1.32
USP7	NM_003470	4.90*E* − 04	1.39
PHKB	NM_000293	5.07*E* − 04	1.28
MED1	NM_004774	5.19*E* − 04	1.41
NDUFA5	NM_005000	5.78*E* − 04	1.51
IKZF1	NM_001220765	1.36*E* − 04	1.56
C7orf43	NM_018275	1.87*E* − 04	1.29
RAB3D	NM_004283	2.51*E* − 04	1.33
CDV3	NM_001134422	2.59*E* − 04	1.25
FRS3	NM_006653	2.64*E* − 04	1.23
ARF3	NM_001659	3.46*E* − 04	1.34
LMO4	NM_006769	3.64*E* − 04	1.22
RING1	NM_002931	4.61*E* − 04	1.23
NADK	NM_001198993	5.82*E* − 04	1.36
MTMR3	NM_001013676	5.99*E* − 04	1.28
PTGES2	NM_001256335	6.04*E* − 04	1.34
MAX	NM_001271068	6.32*E* − 04	1.47
LOC100996752	NM_019094	6.73*E* − 04	1.22
MARS	NM_004990	8.23*E* − 04	1.21
SIGLEC7	NM_001277201	8.49*E* − 04	1.24
DUSP3	NM_004090	8.91*E* − 04	1.39
TNFAIP2	NM_006291	9.11*E* − 04	1.38
MAT2A	NM_005911	9.78*E* − 04	1.41
BRD2	NM_001113182	1.06*E* − 03	1.44
MED12	NM_005120	1.23*E* − 03	1.23
SLC25A28	NM_031212	1.26*E* − 03	1.24
STX16	NM_001001433	1.44*E* − 03	1.39
USP21	NM_001014443	1.50*E* − 03	1.26
TM9SF4	NM_014742	1.55*E* − 03	1.39
CBFB	NM_001755	1.57*E* − 03	1.28
GGA2	NM_015044	1.60*E* − 03	1.21
POLG	NM_001126131	1.61*E* − 03	1.34
YKT6	NM_006555	1.68*E* − 03	1.25
CLASRP	NM_001278439	1.75*E* − 03	1.28
RCN3	NM_020650	1.94*E* − 03	1.21

**Table 2:** The top 30 GO terms.

GO ID	GO name	DEG number	*P* value	FDR	Gene symbols
GO:0044281	Small molecule metabolic process	121	1.65*E* − 34	4.62*E* − 31	PPP1CB|PLCB1|CALM1|PIK3CD|PIK3R1|PIK3CA|PTEN|LYPLA1|AMD1|SACM1L|ARF3| INSIG1|PDK3|ABHD5|CHKB|IMPA1,…
GO:0007165	Signal transduction	100	1.19*E* − 31	1.66*E* − 28	IL7R|TNFSF12|TNFSF10|TNFSF4|PPP2R5C|PLCB1|CALM1|PIK3CB|PIK3CD|CASP1| TLR1| RAC2|STAT6|MAPK14|CAMK1|PAK2,…
GO:0045087	Innate immune response	64	1.57*E* − 24	1.47*E* − 21	CASP1|ATF1|PIK3CA|PIK3R1|MAP3K7|MAP3K5|KRAS|TLR1|PTEN|
ATF2|CFP|PTPN11| IRF7|ACTB|CALM1,…					
GO:0010467	Gene expression	69	1.53*E* − 23	1.07*E* − 20	TNFSF13|PPP2R1A|RPL38|MAPK14|KARS|SMAD2|AIMP1|DICER1,…
GO:0006915	Apoptotic process	66	5.32*E* − 22	2.98*E* − 19	IL1A|TNFSF10|TNFSF12|MAP3K7|CASP1|BAX|RAD21|DICER1|CASP4|PTEN|H1F0|FXR1|PSMD12|DAB2|CST3,…
GO:0000278	Mitotic cell cycle	47	2.64*E* − 20	1.23*E* − 17	PPP1R12A|PPP1CB|PPP2R1A|NSL1|RFC5|RAD21|RBL2|RPA1|CEP63…
GO:0006351	Transcription, DNA-dependent	114	1.32*E* − 19	5.28*E* − 17	MAP3K7|ATF1|ATF6|STAT6|FOSL2|ASF1A|BBX|UCHL5|ATXN1|INO80 B|KANK2|CEBPE|SIRT1| SETD2|IRF5| BRD2|IKZF1|RB1,…
GO:0019221	Cytokine-mediated signaling pathway	27	1.63*E* − 19	5.70*E* − 17	IL1A|IL6R|STAT2|STAT6|KRAS|CEBPA|PTPN11|CD44|IRF7|IRF5|IRF3|IRAK3|ZC3H15|CCR1,…
GO:0019048	Virus-host interaction	42	4.74*E* − 19	1.48*E* − 16	MAP3K5|PIK3R1|KARS|BAX|TGFB1|CCR3|MDFIC| RB1|SIRT1,…
GO:0006468	Protein phosphorylation	43	2.25*E* − 18	6.30*E* − 16	MAP3K11|MAP4K4|PIK3R1|PIK3CA|TGFB1|ADAM10|IRAK3|HIPK3|SMAD2| TLK1|CREB1|FYB|STK11,…
GO:0008380	RNA splicing	34	3.49*E* − 16	7.52*E* − 14	PPP2R1A|SRSF11|PABPN1|CPSF1|SRSF3|CLASRP,…
GO:0015031	Protein transport	43	3.87*E* − 16	7.73*E* − 14	RAB3D|ARRB2|RAB11A|ACAP1|NMD3|ARF3,…
GO:0044267	Cellular protein metabolic process	45	4.71*E* − 13	6.94*E* − 11	ATF1|RPL38|ACTB|ATF6|CPA3|EIF4B|ETF1|SPCS2|CXXC1|ALG5|CCT6A,…
GO:0000090	Mitotic anaphase	24	4.61*E* − 12	5.87*E* − 10	PPP2R1A|RAD21|PDS5A|PSMD3|SMC3|CLASP2,…
GO:0000087	M phase of mitotic cell cycle	26	7.27*E* − 12	8.85*E* − 10	PPP2R1A|PSMD4|CLASP2|PSME3|ANAPC5|RAD21|RANBP2,…
GO:0000398	mRNA splicing via spliceosome	24	7.84*E* − 12	9.15*E* − 10	SRSF11|PABPN1|CLP1|RBM22|PAPOLA|FUS|POLR2E|TRA2A|PRPF4B,…
GO:0001525	Angiogenesis	24	9.13*E* − 12	1.02*E* − 09	TNFSF12|MAP3K7|MAPK14|PIK3CA|ELK3|DICER1|SIRT1,…
GO:0002224	Toll-like receptor signaling pathway	19	3.87*E* − 11	3.87*E* − 09	MAP3K7|MAPK14|TLR7|IRF7|ATF1|ATF2|DUSP3|IRF3|HSP90B1|RPS6KA5|BTK| UNC93B1|ELK1|BCL10|UBE2D1,…
GO:0007049	Cell cycle	24	4.71*E* − 11	4.55*E* − 09	PPP1CA|CAMK1|RB1CC1|CREBL2|PARD6A|CYLD|TLK1| IKZF1,…
GO:0006955	Immune response	29	7.21*E* − 11	6.73*E* − 09	IL1A|IL7R|IL1RN|TNFSF10|TNFSF4|TNFSF13|TLR1|CCR1|IKBKAP,…
GO:0008283	Cell proliferation	29	5.42*E* − 10	4.27*E* − 08	IL1A|RBL38|MAP3K11|PTEN|DAB2|FES|PA2G4|ASCC3|MS4A2,…
GO:0030155	Regulation of cell adhesion	10	5.49*E* − 10	4.27*E* − 08	PPP2R1A|PPP1R12 A|PPP1CB|CYTH4|PTK2B| CYTIP| ENG|GSN,…
GO:0050852	T-cell receptor signaling pathway	16	6.55*E* − 10	4.96*E* − 08	MAP3K7|PIK3CA|PIK3CB|PIK3R1|PIK3CD|RBCK1|PAK2|FYB|UBE2N|PTEN|WAS|FYN|NCK1|MALT1|CD4| BCL1
GO:0043065	Positive regulation of apoptotic process	20	8.03*E* − 10	5.92*E* − 08	TNFSF10|PPP2R4|MAP3K5|PIK3R1|TGFB1|ATM|SIRT1|DNM2|LILRB1|CTNNB1|TSPO|HMGB1|BAX|ING3|IRF5|PRKDC,…
GO:0051403	Stress-activated MAPK cascade	10	1.92*E* − 09	1.38*E* − 07	MAPK14|MAP3K7|ATF1|ATF2|ELK1|DUSP3|RPS6KA3|CREB1|RPS6KA1|RPS6KA5
GO:0007010	Cytoskeleton organization	10	4.27*E* − 09	2.49*E* − 07	PRPF40 A|PHIP|SIPA1|PLD2|SVIL|PXN|TPM1|ZMYM4|CAPZB,…
GO:0050900	Leukocyte migration	14	6.19*E* − 09	3.54*E* − 07	PIK3CB| PIK3CA|PIK3R1|KRAS|SIRPA| PTPN11|FYN|ITGB7,…
GO:0006954	Inflammatory response	19	1.11*E* − 08	6.21*E* − 07	IL1A|PIK3CD|TLR1|TLR7|STAB1|TGFB1|HRH4|CCR3|CCR1|LTB4R,…
GO:0008219	Cell death	16	8.44*E* − 08	3.94*E* − 06	MAP3K11|BCL10|FOSL2|CLN3|ATXN7|DCTN1|FUS|,…
GO:0006281	DNA repair	30	9.06*E* − 08	4.16*E* − 06	BTG2|RFC5|SMC3|RPA1|TDG|CSNK1D|REV1,…

**Table 3:** Results of pathway enrichment analysis of DEGs.

Pathway name	DEG number	*P* value	Gene symbols
Osteoclast differentiation	26	6.26*E* − 16	TYK2|PIK3CA|PIK3CB|PIK3CD|PIK3R1|IL1A|PPP3CA|JUND|FOSL2|MAPK14|MAP3K7|STAT2|TGFB1,…
Metabolic pathways	79	2.81*E* − 15	COX11|PIP5K1A|PLCB1|CHPF2|ALOX5|TPK1,…
PI3K-Akt signaling pathway	34	1.77*E* − 11	IL7R|IL6R|PPP2R5C|PPP2R1A|PIK3CB|PIK3CD|PIK3CA|ATF2,…
MAPK signaling pathway	28	1.34*E* − 10	IL1A|PPP3CA|JUND|MAPK14|MAP3K5|MAP3K7|KRAS|TGFB1,…
Chemokine signaling pathway	24	1.39*E* − 10	PLCB1|CCR1|CCR3|PIK3R1|PIK3CD|PIK3CB|PIK3CA|KRAS|RAC2|STAT2,…
T-cell receptor signaling pathway	17	2.29*E* − 09	PPP3CA|MAPK14|MAP3K7|PIK3CD|PIK3CB|PIK3R1|PIK3CA|KRAS|BCL10,…
Focal adhesion	23	3.28*E* − 09	PPP1CA|PPP1CB|ACTB|PIK3CA|PIK3CD|PIK3CB|PTEN,…
Apoptosis	15	6.96*E* − 09	IL1A|TNFSF10|TNFRSF10B|PIK3CD|PIK3CB|PIK3CA|BAX|IRAK3,…
Pathways in cancer	27	9.27*E* − 08	PIK3CA|PIK3CB|PIK3R1|PIK3CD|KRAS|PTEN|MAX|RAC2,…
Cell cycle	16	1.29*E* − 07	ANAPC5|TGFB1|SMAD2|ATM|RBL2|RB1,…
VEGF signaling pathway	11	1.26*E* − 06	PPP3CA|MAPK14|PIK3CA|PIK3CD|PIK3R1|PIK3CB|RAC2|KRAS,…
Toll-like receptor signaling pathway	11	1.37*E* − 04	MAPK14|MAP3K7|PIK3CD|PIK3R1|PIK3CA|PIK3CB|TLR7|TLR1|IRF5|IRF7,…
Calcium signaling pathway	14	3.47*E* − 04	PPP3CA|PLCB1|CALM1|ITPR1|ITPR2|PTK2B,…
p53 signaling pathway	8	4.91*E* − 04	TNFRSF10B|ATM|PTEN|THBS1|BAX|CCNG2|CCNG1,…
Wnt signaling pathway	10	5.49*E* − 03	PPP3CA|MAP3K7|PLCB1|RAC2|CHP|,…
TGF-*β* signaling pathway	7	7.29*E* − 03	PPP2R1A|TGFB1|SMAD2|SMURF2,…
Jak-STAT signaling pathway	10	1.11*E* − 02	IL7R|IL6R|PIK3R1|PIK3CA|PIK3CD|PIK3CB|STAT2|STAT6,…

## Data Availability

The data used to support the findings of this study are available from the corresponding author upon request.

## Abbreviations

OA: Osteoarthritis
PBMCs: Peripheral blood mononuclear cells
IL-1RI: Interleukin 1 receptor type I
MMP13: Matrix metallopeptidase 13
DEGs: Differentially expressed genes
GO: Gene ontology
GCBI: Gene-Cloud of Biotechnology Information
ACR: American College of Rheumatology
RA: Rheumatoid arthritis
*RPL38*: Ribosomal protein L38
*PPP3CA*: Protein phosphatase 3, catalytic subunit, alpha isozyme
*IKZF1*: IKAROS family zinc finger 1
*C7orf43*: Chromosome 7 open reading frame 43
*IL1A*: Interleukin 1, alpha
*IL6R*: Interleukin 6 receptor
*IL7R*: Interleukin 7 receptor
*PIK3*: Phosphoinositide-3-kinase
*MAPK14*: Mitogen-activated protein kinase 14
*TNFSF10*: Tumor necrosis factor (ligand) superfamily, member 10
*TNFSF13*: Tumor necrosis factor (ligand) superfamily, member 13
*ATF2*: Activating transcription factor 2
*TYK2*: Tyrosine kinase 2
*PIK3CA*: Phosphatidylinositol-4,5-bisphosphate 3-kinase, catalytic subunit alpha
*PIK3CB*: Phosphatidylinositol-4,5-bisphosphate 3-kinase, catalytic subunit beta
*PIK3CD*: Phosphatidylinositol-4,5-bisphosphate 3-kinase, catalytic subunit delta
*PIK3R1*: Phosphoinositide-3-kinase, regulatory subunit 1 (alpha);
*MAPK14*: Mitogen-activated protein kinase 14
*JUND*: Jun D proto-oncogene
*FOSL2*: FOS-like antigen 2.
